# Efficacity of CT-guided intra-articular cervical facet steroid injection for cervical radiculopathy

**DOI:** 10.1016/j.redii.2024.100050

**Published:** 2024-06-11

**Authors:** Clément Ravenel, Charlotte Martin-Peltier, Maxime Lacroix, Fadila Mihoubi-Bouvier, Christelle Nguyen, Romain Touzé, Jean-Luc Drapé

**Affiliations:** a Service radiologie B, hôpital Cochin, AP-HP, Centre-Université Paris Cité, 27, rue du Faubourg-Saint-Jacques, 75014 Paris, France; b UFR de médecine, faculté de santé, Université Paris Cité, 75006 Paris, France; c Service de rééducation et réadaptation de l'appareil locomoteur et des pathologies du rachis, hôpital Cochin, AP-HP, Centre-Université Paris Cité, 75014 Paris, France; d UMR-S 1124 Toxicité environnementale, cibles thérapeutiques, signalisation cellulaire et biomarqueurs (T3S), Inserm, campus Saint-Germain-des-Prés, 75006 Paris, France; e Service d'ophtalmologie, hôpital Necker-Enfants Malades, AP-HP, Centre-Université Paris Cité, 75006 Paris, France; f ECaMO Team, UMR-S 1153, Centre de recherche épidémiologie et statistique (Cress), Inserm, 75004 Paris, France

**Keywords:** Cervical radiculopathy, Intra-articular facet steroid injection, CT guidance

## Abstract

**Background:**

Traditionally, transforaminal steroid injection is performed in the management of cervical radiculopathy in medical failure treatment but carried a true risk of catastrophic complication. Another approach currently used is to perform intra-articular facet steroid injection to reach the epidural space.

**Purpose:**

The aim of this study was to describe the evolution of symptoms following intra-articular facet steroid injection in cervical radiculopathy.

**Material and methods:**

We conducted a retrospective study. We assessed all patients who had a CT-guided intra-articular facet steroid injection in our center (xx, xx, xx) from December 2015 to February 2021. Cervical MR pretherapeutic images were analyzed and classified according to cervical pain etiology: uncodiscarthrosis, disk herniation or congestive cervical posterior osteo-arthritis. All patients had clinical initial evaluation and then follow-up at 1 and 6 months. Pain severity was rated on a visual analog scale and expressed as a percentage of improvement.

**Results:**

Ninety-three patients were included. There were 56 patients with uncodiscarthrosis, 29 with a disk herniation and 8 with a cervical posterior congestive osteoarthritis. A significant improvement of the visual analog scale percentage was found for all patient at 1 and 6 months (*p* < 0.01). Visual analog scale percentage improvement was about 50 % for all etiologies. For all patients, no severe complications were reported.

**Conclusion:**

Intra-articular facet steroid injection may be considered for the treatment of cervical radiculopathy when other medical treatments have failed.

## 1 Introduction

Cervical radiculopathy is a frequent and common pathology that involves pain perceived in the neck with radiation to the upper extremity [1]. This clinical condition results from the compression of a cervical root or an inflammation process surrounding the root. In most cases, it affects lower cervical spine (from C4 to C7) [2]. Common causes are differentiated from symptomatic causes (hematoma, infection and inflammatory pathology). The two main etiologies of common cervical radiculopathy are uncodiscarthrosis and disc herniation [3]. Uncodiscarthrosis is the most frequent (70 %) [4]. Another under-studied cause is congestive posterior articular osteoarthritis. Indeed, 80 % of congestive flare-up of posterior articular osteoarthritis induce an ipsilateral cervical radiculopathy. Prevalence of congestive posterior osteoarthritis in CR is about 10 % [5].

Non-invasive management includes relative rest, oral analgesics, non-steroidal anti-inflammatory drugs and physical therapy with inconclusive evidence for their effectiveness [6,7]. For patients with cervical radiculopathy, improvements in pain and function tend to occur only within several months, with significant disability and economic impact [2]. In case of failure of these treatments, image-guided nerve root infiltration of steroids can be performed. However, surgery does not have any place in first line; indeed, the benefit / risk balance is low with a high risk of complication [8]. Traditionally, transforaminal steroid injection (TFSI) or even interlaminar epidural injection were used. Theoretically, TFSI is supposed to deliver a selective and higher concentration of steroids near the cervical nerve root and in the ventral epidural space [9,10]. Nevertheless, TFSI seems to be responsible for extremely rare but catastrophic complications. Reports of quadriparesis, stroke and even death following TFSI began appearing in the early 2000s [11]. Intra-articular facet steroid injection (IFSI) is an alternative technique available to deliver steroids close to the cervical nerve root. Indeed, the cervical posterior facet joint is a way of diffusion to the foraminal space, through the retrodural space also called space of Okada [12]. This method, little studied in the literature, seems interesting because it is technically easier and presents fewer neurological risks. A recent study showed an equivalent efficacity of IFSI and TFSI in this indication, but there were some limits, in particular, the clinical outcomes were only evaluated early at one month [13]. Another study demonstrated the efficacity of IFSI but the infiltrations were performed with fluoroscopy [14], which induces a risk of navigation error. Moreover, in these two studies the cause of the cervical radiculopathywas not investigated. Therefore, this study aimed at evaluating the efficacity of IFSI with CT guidance in patients with cervical radiculopathy. A secondary objective was to better identify the effectiveness of ISFI according to the etiology.

## 2 Material and methods

### 2.1 Participants

We conducted a single-centered retrospective study. We identified all patients who underwent an IFSI from December 2015 to February 2021 in our center (xx, xx, xx) using our local pictorial archive and communication system (Carestream 3.2, Carestream Health, Rochester, New York). All patients gave oral consent before IFSI. To ensure full consistency of the level injected and the most plausible nociceptive lesion, all patients had a consultation in a multidisciplinary team meeting, during which the clinical presentation and imaging were reviewed by a multidisciplinary team of spine specialists (i.e. radiologists, rheumatologists, spine surgeons, physiatrists) with at least 5-year experience in the management of spine disorders. If several anatomical lesions were present, the patient was classified in the group of the lesion most consistent with the clinical presentation and that was considered for injection. For the purpose of our study, patients with mixed lesions (i.e., combination of uncarthrosis, disk herniation and/or posterior impingement due to a congestive posterior articular) at the same level were not included. The eligibility criteria were patients over 18 years old with a common cervical radiculopathy who underwent IFSI under CT guidance in our center. Only patients with a single site of injection were included. We excluded patients who did not perform cervical MR in the preceding six months, or who did not get complete follow-up (at least 6 months).

### 2.2 MR analysis

**Fig. 1:**
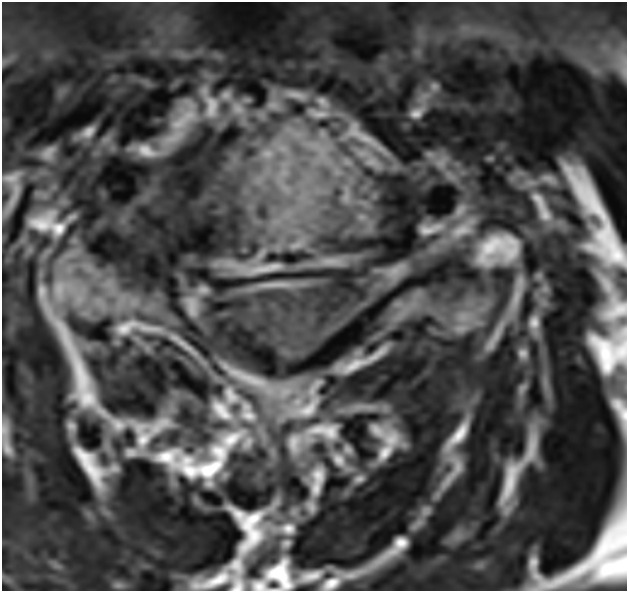
Study on intra-articular facet steroid injection in cervical radiculopathy. Group A was defined by the presence of osteoarthritic changes involving the uncus. Severe right uncodiscarthrosic stenosis at the C4-C5 level. Axial 3-dimension T2-weighted sequence.

**Fig. 2:**
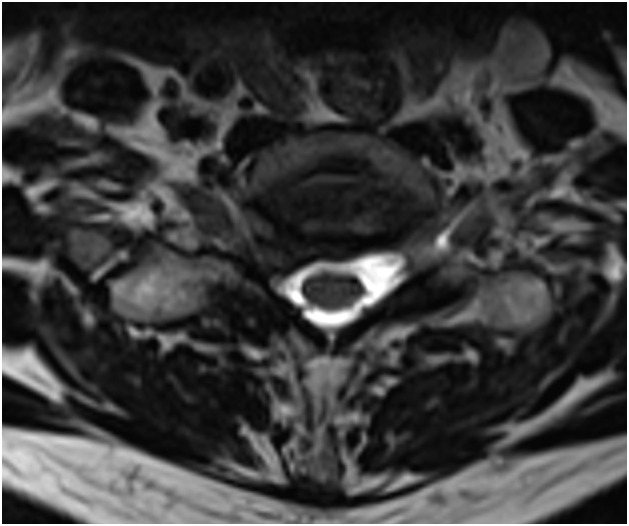
Study on intra-articular facet steroid injection in cervical radiculopathy. Group B was defined by the presence of an herniated disc. Right foraminal disc protrusion at the C5-C6 level. Axial 3-dimension T2-weighted sequence.

**Fig. 3:**
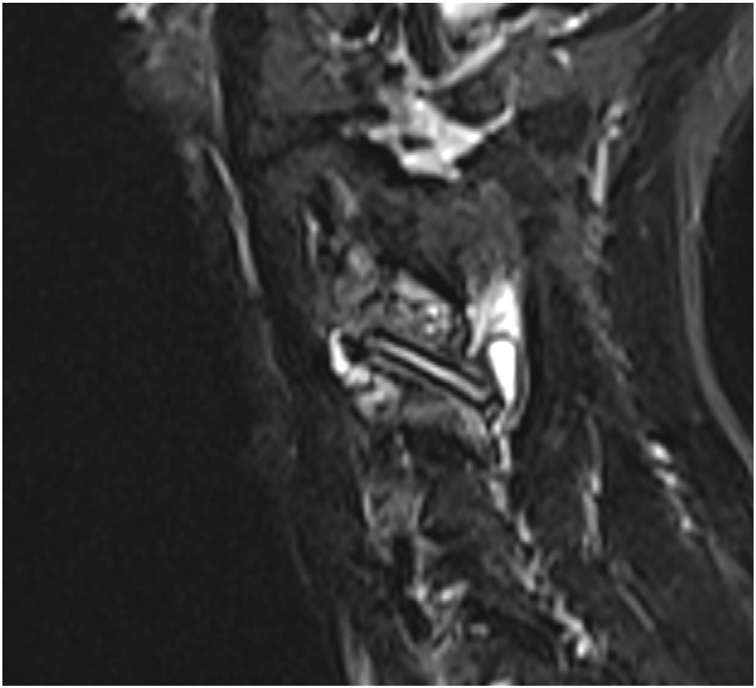
Study on intra-articular facet steroid injection in cervical radiculopathy. Group C was defined by the presence of congestive osteoarthritic changes involving the facet joint. Right C3-C4 congestive facet joint osteoarthritis. Bone marrow edema and intra-articular effusion. T2-Dixon Water weighted parasagittal sequence.

Cervical MR images were analyzed on our institutional imaging server. Examinations were not all performed in our center, so MR imaging protocol was not similar for all patients. A single junior radiologist with one year of experience in musculoskeletal imaging reviewed all exams. MR imaging all contained at least a sagittal T2-weighted sequence, a sagittal T1-weighted and an axial T2-weighted acquisition. Most examinations specifically encompassed an inflammatory sequence (T2-weighted sequence with fat suppression technique), such as a sagittal T2-Dixon, sagittal STIR or coronal STIR. MR imaging was reviewed to confirm the presence of uncodiscarthrosis, disk herniation or congestive posterior articular osteoarthritis at the level of the involved cervical nerve. Three different groups were defined according to the presence of anatomical changes on MRI including at least a sagittal T2-, a sagittal T1- and an axial T2-weighted sequence, consistent with clinical presentation, as assessed during a multidisciplinary team meeting. Group A (Fig. 1) was defined by the presence of osteoarthritic changes involving the uncus, group B (Fig. 2) by the presence of a herniated disc and group C (Fig. 3) by the presence of congestive osteoarthritic changes involving the facet joint. When an inflammatory sequence was missing, congestive posterior osteoarthritis changes were defined as facet joint osteoarthritic changes associated with joint hypointense signal in sagittal T1-weighted sequence and hyperintense signal in sagittal T2-weighted sequence.

### 2.3 Intervention

**Fig. 4:**
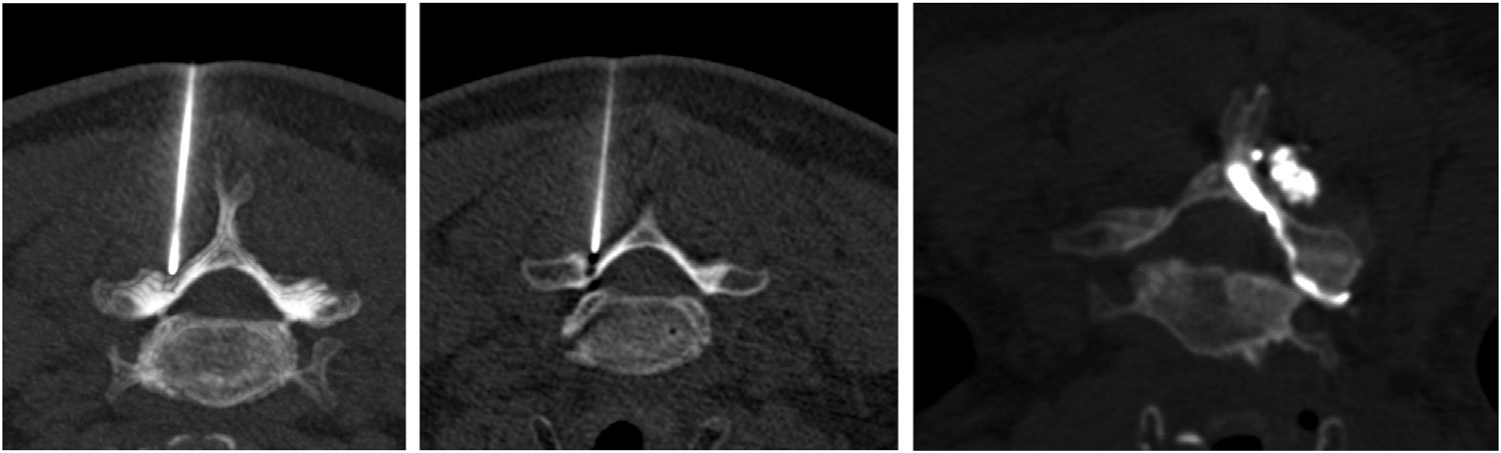
Study on intra-articular facet steroid injection in cervical radiculopathy. Correct position of the needle and correct opacification by the contrast agent. (Left) Positioning of the needle in the posteroinferior recess of the joint. Image scan axial MIP. (Middle) Same image without MIP. (Right) Axial can after infiltration with opacification of the facet joint and passage of contrast in the epidural space of Okuda.

The intervention was realized by three radiologists from our center, all with experience in musculoskeletal imaging ranging from 3 to 15 years. We performed a checklist safety before all interventions: iodine-based contrast allergy, current infection, coagulopathy and absence of cervical surgery. All patients were installed in the prone position. The injection was performed in sterile conditions by a standardized technique [15] under CT guidance (Somaton Definition Flash, Siemens, Germany). The appropriate entry site was marked on the skin after spotting. Then a 22-ga 3,5-inch spinal needle was advanced by using intermittent CT-controlled. Once the needle was in the appropriate location, 0.5–1 ml of iodinated contrast in the cervical facet joint was injected. The iodinated contrast injected was Visipaque 320 mg iodixanol/ml. The foraminal space could be opacified in some cases after contrast injection in the cervical facet joint (Fig. 4). A final CT-control was then performed to exclude an intra-vascular position and to confirm the adequate intra-articular distribution of the contrast. Then, 1 ml of dexamethasone sodium phosphate 10 mg/ml was injected, and the needle was withdrawn. The infiltration was performed on the ipsilateral posterior cervical facet of the cervical radiculopathy. No local anesthetics were used during the procedure. The patient was systematically observed for 30 min after the intervention.

### 2.4 Outcomes

The indication of injection and the follow-up were performed by physicians from the physical medicine and rehabilitation department of our center. The clinical outcome measure was pain severity rated on a visual analog scale (VAS): defined by a line extending from 0 (no pain) to 10 (worst pain imaginable). All patients were evaluated with this VAS three times by physicians: before the intervention, then 0-1 month and 1–6 months after the intervention. Pain assessments before and after could be performed either by the treating physician and/or by the radiologist, in the context of health care. The improvement of the pain compared to the initial consultation was expressed using a percentage improvement of the VAS (VAS%). VAS% was calculated in the first month after the infiltration (compared with the initial consultation) and between 1 and 6 months after the infiltration (compared with the initial consultation too). For all patients, we also specified if they took analgesic treatment including morphine and if they did or did not have a professional activity during their pathology (classical factors predictors of poor response). Patients in retirement were considered in the professional activity group. Complications were also noted: hematoma, infection and neurological accident. Clinical outcomes were analyzed in blind of the MRI results.

### 2.5 Statistical analysis

The data analyses were performed with RStudio software (RStudio, Inc, version 1.2.5033). Quantitative continuous variables results were presented as means with standard deviation or median with range according to the distribution and qualitative variable by frequency (%). The normal distribution of variables was tested using a Shapiro test. Quantitative variables were tested using a Wilcoxon test or a student *t*-test according to their distribution. For analyses with more than two groups, a one-way ANOVA analysis was used with Tukey's multiple comparisons. For all analyses, significant results were obtained by *p* < 0.05.

### 2.6 Ethical consideration

Because our research was retrospective, based on data already collected in the context of health care, which therefore, by definition, did not involve human beings directly, it did not fall under the Jardé Law of March 5th, 2012 and its application decree (No. 2016-1537) related to research involving the human person in France (https://www.legifrance.gouv.fr/jorf/id/JORFTEXT000025441587, accessed on February 8th, 2023). Therefore, a formal approval from an Institutional Review Board (IRB) is not mandatory. Further, in our experience, requesting an authorization of an ethical committee (e.g., Cerim), after the research was conducted, is usually considered as inappropriate by the committees. For this reason, we did not request this authorization for the purpose of the present manuscript. Finally, all patients, in the context of health care offered in our institution, were fully informed that data could be retrieved from their medical records for research purpose and were free to decline at any time.

## 3 Results

### 3.1 Participants

Overall, 401 patients with intra-articular cervical facet joint steroid infiltrations were initially eligible; 308 patients were excluded: 110 did not have a complete clinical follow-up and 198 did not have MR imaging available or achieve within the 6 months. Finally, 93 patients were included in this study (Table 1). It was the first IFSI for all participants.

**Table 1:** Study on intra-articular facet steroid injection in cervical radiculopathy: Demographical and clinical characteristics of the analyzed population and site of injection.

	Total *N* = 93	Group A *N* = 56	Group B *N* = 29	Group C N = 8
*Demographical*				
Patients	93	56 (60 %)	29 (31 %)	8 (9 %)
Male/Female	42/51	26/30	13/16	3/5
Median age (years)	55	57	47	67
*Clinical*				
Morphine intake	14/93 (15 %)	8/56 (14 %)	6/29 (21 %)	0/8
Patient in professional activity	71/93 (76 %)	44/56 (79 %)	19/29 (66 %)	8/8 (100 %)
*Site of injection*				
C2-C3	1	0	0	1
C3-C4	5	2	0	3
C4-C5	5	2	1	2
C5-C6	45	25	19	1
C6-C7	32	24	8	0
C7-T1	5	3	1	1

Group A: impingement due to uncarthrosis.

Group B: impingement due to disk herniation.

Group C: impingement due to congestive posterior articular.

Demographical and clinical characteristics of the analyzed population and site of injection are reported in Table 1. Only one had an infiltration at the upper cervical spine (floor C2-C3), for a congestive posterior articular osteoarthritis. A “complete” MRI (with an inflammatory sequence) was available for 58 patients: 19 patients with a coronal STIR acquisition and 39 patients with a sagittal T2-Dixon or STIR acquisition.

### 3.2 Outcomes

**Fig. 5:**
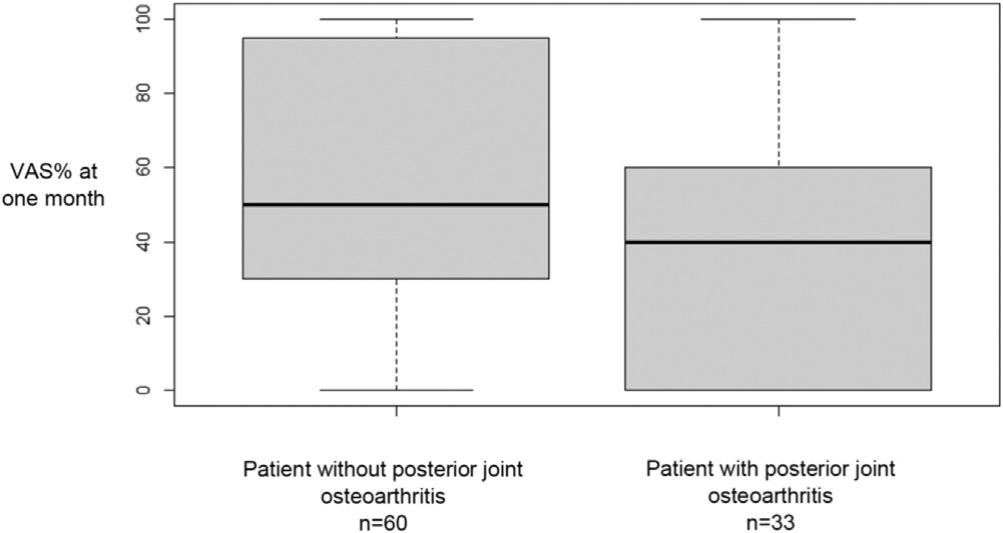
Study on intra-articular facet steroid injection in cervical radiculopathy: Evaluation of visual analog scale percentage (VAS%) at 1 month according to posterior joint osteoarthritis. *p* = 0.04.

**Fig. 6:**
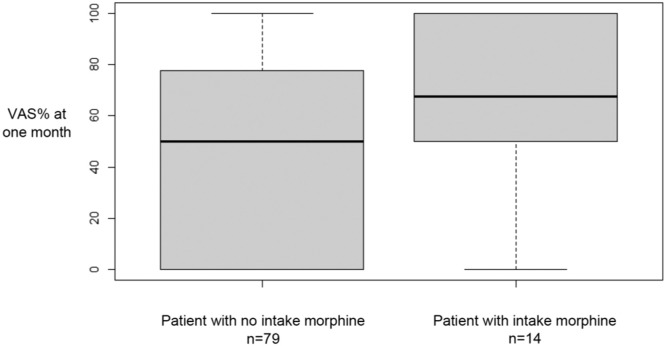
Study on intra-articular facet steroid injection in cervical radiculopathy: Evaluation of visual analog scale percentage (VAS%) at 1 month according to intake morphine. *p* = 0.1.

**Fig. 7:**
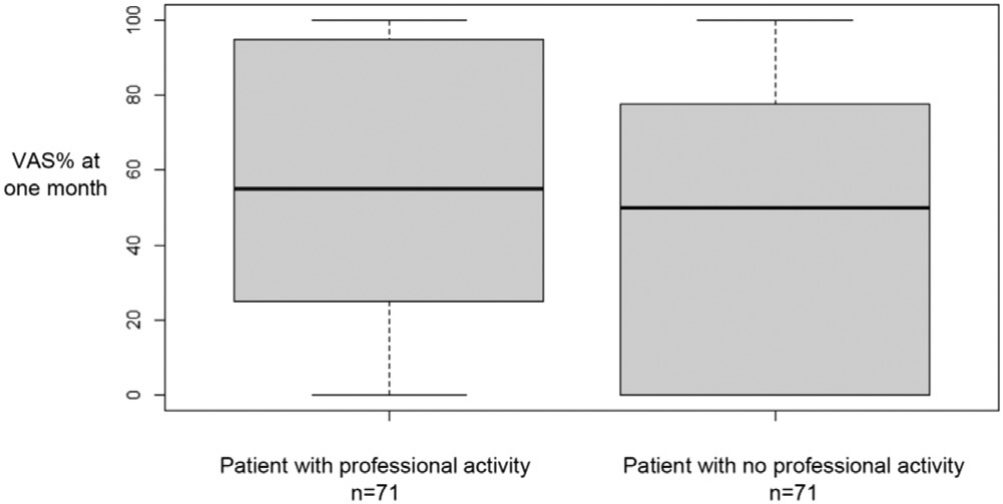
Study on intra-articular facet steroid injection in cervical radiculopathy: Evaluation visual analog scale percentage (VAS%) at 1 month according to professionnal activity. *p*1 = 0.35.

For all patients, we found a significant improvement of the VAS% after 1 month and 6 months (Table 2). We also found a significant improvement of the VAS% for each subgroup at 1 month and 6 months (Table 2). In fact, we found an average improvement of the VAS% about 50 % VAS% seemed to be lower at 6 months than at 1 month, but this difference was not statistically significant (Table 2). The percentage of patients who experienced less than 30 % of% VAS after IFSI was 37 % at 1 month and 47 % from 1 to 6 months. There was no significant difference at 1 month and at 6 months of VAS% between the three subgroups (Table 3). VAS% at 1 month was significantly better in patients without posterior joint osteoarthritis (Fig. 5). However, there was no significant result concerning the morphine intake and the absence of professional activity (Fig. 6, Fig. 7). For all patients, no severe complications occurred or were reported immediately or in the days following the procedure.

**Table 2:** Study on intra-articular facet steroid injection in cervical radiculopathy: Visual analogue scale (VAS) amelioration after injection at 1 month and 6 months after.

	VAS% 1 month	VAS% 6 months	*p* (ANOVA)	*p* VAS% initial vs. VAS% 1 month	*p* VAS% initial vs. VAS% 6 months	*p* VAS% 1 month vs. VAS% 6 months
Total	48.9 %	44.7 %	<0.01	<0.01	<0.01	0.24
Group A	48.8 %	44.9 %	<0.01	<0.01	<0.01	0.56
Group B	49.3 %	44.3 %	<0.01	<0.01	<0.01	0.43
Group C	48.7 %	45.0 %	<0.01	0.02	0.04	0.85

Group A: impingement due to uncarthrosis.

Group B: impingement due to disk herniation.

Group C: impingement due to congestive posterior articular.

**Table 3:** Study on intra-articular facet steroid injection in cervical radiculopathy: Comparison of the improvement in the visual analogue scale (VAS) after injection at 1 and 6 months according to the type of impingement.

	**Group A**	**Group B**	**Group C**	***p* (ANOVA)**	***p* Conflict ant vs. Conflict post**	***p* Conflict ant vs. Articular post**	***p* Conflict post vs. Articular post**
**VAS% 1 month**	48.8 %	49.3 %	48.7 %	0.99	0.99	0.99	0.99
**VAS% 6 months**	44.9 %	44.3 %	45.0 %	0.99	0.99	0.99	0.99

Group A: impingement due to uncarthrosis.

Group B: impingement due to disk herniation.

Group C: impingement due to congestive posterior articular.

## 4 Discussion

The main result of this study is that IFSI is effective in reducing pain in patients with cervical radiculopathy at 1 month and 6 months. This result was found for all causes of cervical radiculopathy, even if the most common etiology in our study was uncodiscarthrosis.

IFSI is a technique used in daily practice despite poor literature. In our study, VAS% was about 50 % in all configurations. This result is similar with literature: in fact, Bureau and al. found 45 % of VAS% improvement using IFSI [13]. But the evaluation was done on 28 patients and only performed at one month. Also, Kelekis et al. in 2014 studied retrospectively the safety and efficacy of percutaneous fluoroscopically guided nerve root infiltrations in cases of cervical radiculopathy using an indirect postero-lateral facet approach. They found a decrease of 6.96 on the numeric visual scale (NVS) unit, but after a mean of 2.3 sessions of infiltration for each patient [14].

The other main non-surgical technique for the support of cervical radiculopathy in medical treatment failure is TFSI. A recent meta-analysis found an improvement about 50 % of the VAS at 3 months by using this technique in patients with a cervical radiculopathy [16]. More anecdotally, using intra-discal cervical steroid injection in patients with a cervical radiculopathy due to a cervical uncodiscarthrosis also allow an improvement about 50 % of the VAS% at 6 months [17]. Indeed, cervical radiculopathy is not a current indication for intradiscal infiltration. Thus, according to the literature IFSI seems to be as equal in efficacity as the other non-surgical treatment.

In our study we did not investigate the distribution pattern of the contrast product, but Bureau et al. in a retrospective study, found juxta-articular spreading contrast as a predictor of therapeutic effectiveness during an IFSI [12]. We did not succeed to highlight a significant difference in VAS% between the three groups, it is probably due to our small sample and to the EVA scale (0–10) used not very discriminating. Some articles showed that IFSI appeared to be more effective in CR due to a disk herniation [13]. It might be interesting to prove this result with a larger sample and a more relevant clinical evaluation. The presence of degenerative posterior osteoarthritis was a significant predictive factor of poor response and could be explained by per-procedure difficulties to reach the intra-articular facet space and therefore to allow a good diffusion of the steroid in the retrodural and foraminal space. In our study values of VAS% in all groups seemed lower at 6 months versus at 1 month, even if this difference is not statistically significant, this could be due to an initial placebo effect. Thus, it could be interesting to evaluate this effect over a longer period time.

There were several limitations to the present study, first including those inherent in any retrospective study. There was modest sample size, especially with a small sample of congestive cervical articular posterior. The size of our sample did not permit to obtain a sufficient power to compare the three groups and some sub-groups (such as morphinic intake or professional activity). Furthermore, all patients did not have the same MR sequences they did not all have an inflammatory sequence which may have under-estimated the prevalence of congestive posterior articular. Also, images were not necessary acquired on the same MR device and an evaluation of the diffusion of the contrast material was not performed. Several operators carried out the infiltration and one cannot exclude that operators’ experience may have influenced outcomes. However, we did not specifically collect the percentage of IFSI performed by each radiologist for the purpose of the present study and did not analyze the difference between the results of the three operators (junior versus senior). Finally, one cannot exclude that response may be different between male and female patients. However, we did not collect this information and did not prespecify a specific analysis.

## 5 Conclusion

IFSI is barely described in literature although it is a common clinical practice in the treatment of cervical radiculopathy. The main result of this study is that IFSI allows an improvement of 50 % in patient with cervical radiculopathy whatever the etiology. This is all the most interesting that no complication occurred in our study, especially neurological complications which are described in TFSI. It could be interesting to confirm this result by a prospective larger study, which could, in particular, better identify the efficacy of IFSI according to the type of impingement and evaluate patients over a longer period. Further, a prospective study comparing IFSI, TFSI and placebo could be interesting.

## Funding

This research did not receive any specific grant from funding agencies in the public, commercial or not-for-profit sectors.

## Availability of data and material (data transparency)

Academic researchers can request access to data and material by contacting Dr. Clément Ravenel at clementravenel@gmail.com.

## Ethical consideration

Because participants’ data were retrospectively retrieved from their medical records for the purpose of the present study, a formal approval by an institutional review board is not required according to the Jardé Law of March 5th, 2012 and its application decree (No. 2016-1537) related to research involving the human person in France (https://www.legifrance.gouv.fr/jorf/id/JORFTEXT000025441587, accessed on February 8th, 2023).

## Consent to participate (include appropriate statements)

All participants in the study were informed that information from their medical records could be used for research purpose and did not decline.

## Consent for publication (include appropriate statements)

All authors listed provided final written approval of the version to be published and agreement to be accountable for all aspects of the work in ensuring that questions related to the accuracy or integrity of any part of the work are appropriately investigated and resolved.

## CRediT authorship contribution statement

**Clément Ravenel:** Conceptualization, Methodology, Validation, Formal analysis, Investigation, Data curation, Writing – original draft, Visualization. **Charlotte Martin-Peltier:** Conceptualization, Methodology, Validation, Formal analysis, Investigation, Writing – original draft. **Maxime Lacroix:** Validation, Investigation, Writing – review & editing, Visualization. **Fadila Mihoubi-Bouvier:** Validation, Investigation, Writing – review & editing. **Christelle Nguyen:** Conceptualization, Methodology, Validation, Investigation, Writing – review & editing, Supervision, Project administration. **Romain Touzé:** Validation, Investigation, Writing – review & editing. **Jean-Luc Drapé:** Conceptualization, Validation, Investigation, Writing – review & editing, Supervision.

## Declaration of competing interest

The authors declare that they have no known competing financial interests or personal relationships that could have appeared to influence the work reported in this paper.
